# Flexible Macroblock Ordering for Context-Aware Ultrasound Video Transmission over Mobile WiMAX

**DOI:** 10.1155/2010/127519

**Published:** 2010-08-05

**Authors:** Maria G. Martini, Chaminda T. E. R. Hewage

**Affiliations:** Wireless Multimedia Networking Research Group, Kingston University, London, UK

## Abstract

The most recent network technologies are enabling a variety of new applications, thanks to the provision of increased bandwidth and better management of Quality of Service. Nevertheless, telemedical services involving multimedia data are still lagging behind, due to the concern of the end users, that is, clinicians and also patients, about the low quality provided. Indeed, emerging network technologies should be appropriately exploited by designing the transmission strategy focusing on quality provision for end users. Stemming from this principle, we propose here a context-aware transmission strategy for medical video transmission over WiMAX systems. Context, in terms of regions of interest (ROI) in a specific session, is taken into account for the identification of multiple regions of interest, and compression/transmission strategies are tailored to such context information. We present a methodology based on H.264 medical video compression and Flexible Macroblock Ordering (FMO) for ROI identification. Two different unequal error protection methodologies, providing higher protection to the most diagnostically relevant data, are presented.

## 1. Introduction

The most recent network technologies are enabling a variety of new applications thanks to the provision of increased bandwidth and better management of Quality of Service. Nevertheless, telemedical services involving multimedia data are still lacking behind, due to the concern of the end users, that is, clinicians and also patients, about the low quality provided. This is in particular true in the case of wireless and mobile telemedicine services. Wireless and mobile telemedicine underpins applications such as the transmission of video data from an ambulance, the rapid retrieval and remote display of video data stored in hospital databases, remote (first-level) diagnosis in rural areas, for example, robotic teleultrasonography and telesurgery.

One of the key challenges is the ability to stream medical video over wireless channels. Although wireless multimedia telemedicine services have been proposed before in [1–5], the application of these technologies in real scenarios has been constrained by the unacceptably poor quality of the medical multimedia data arising from the limited bandwidth.

For instance, cardiac ultrasound loops require a very large bandwidth. In diagnostic cardiology it would be desirable to store approximately 30 seconds of dynamic heart images per patient (i.e., three sections of the heart and 10 seconds for each section). Even if frames are digitized with 512 × 512 pixels with 8 bits each and the frame rate is 25 Hz, the size of the uncompressed digital video sequence would be (512 × 512 × 8 × 3 × 10 × 25) bits = 196 M Bytes per examination.

Medical video compression techniques are thus required. For telemedical applications, such techniques must offer high fidelity in order to avoid the loss of vital diagnostic information. To achieve this, lossless compression techniques are often considered, but have the disadvantage of low-compression rates. Therefore, when transmission is over band-limited and error-prone channels, a compromise must be made between compression fidelity and protection and resilience to channel errors and packet loss. It has been estimated that lossy compression ratios of 1 : 5 to 1 : 29 do not result in a lowering of diagnostic accuracy [6]. Furthermore, even in situations where the final diagnosis must be carried out on an image that has been reversibly compressed, irreversible compression can still play a critical role where quick access over a bandlimited channel is required [7].

However, we can consider three types of lossless compression: information lossless compression, perceptually lossless compression, and diagnostically lossless compression. The first one is limited by the entropy (mean information) of the source; the second is such that losses are not perceived by the human eye; the latter is such that the diagnosis made on the basis of the image/video sequence is not affected by compression.

In this paper, our goal is to achieve diagnostic lossless compression and transmission of medical video. We focus on ultrasound video, although some considerations are still valid for different type of sources.

We propose to exploit all the available context information (type and goal of ongoing/stored examination, status of the patient, transmission scenario) to design an appropriate transmission system for diagnostically lossless ultrasound video transmission over WiMAX systems. For instance, coronary heart disease can be diagnosed by measuring and scoring regional motion of the heart wall in ultrasound images of the left ventricle of the heart [8]. Such information can be taken into account as context, and regions of interest (ROI) can be defined accordingly in each specific session. Multiple regions of interest can be defined, and compression/transmission strategies can be tailored to such context information. We propose a global scheme for error-resilient transmission of Ultrasound and ECG data designed based on the characteristics of the specific scenario. For this purpose we invoke a wide range of recent advancements in video compression, error resilient video coding, and transmission technologies, including specific tools of the H.264 video coding standard such as Flexible Macroblock Ordering (FMO) for ROI identification. Two different unequal error protection methodologies, providing higher protection to the most diagnostically relevant data, are proposed and the whole transmission system is specifically designed for the aforementioned scenario. The performance is evaluated over a realistic WiMAX network, by considering real measurements.

The paper is structured as follows. Section 2 provides an overview of the state of the art in wireless telemedicine systems for diagnostic video transmission and also focuses on the main advancements in the relevant enabling technologies. Following the problem statement and description of the proposed approach in Section 3, Section 4 details the system implementation and validation results. Finally, conclusions are drawn in Section 5.

## 2. Main Concepts and State of the Art

### 2.1. WiMAX

In the last few years wireless Metropolitan Area Networks increased momentum. IEEE 802.16/WiMAX (Worldwide Interoperability for Microwave Access) [9–11] is one of the most promising technologies for broadband wireless access, both for fixed and mobile use, currently being deployed in many countries worldwide.

The IEEE 802.16 standard offers broadband wireless access over long distance. Since 2001 WiMAX has evolved from 802.16 to 802.16d for fixed wireless access, and to the new IEEE 802.16e standard with mobility support [9, 10].

The latter is generally referred to as mobile WiMAX. Mobile WiMAX adds significant enhancements, including improvement of NLOS coverage by utilizing advanced antenna diversity schemes and hybrid automatic repeat request (hARQ); the adoption of dense subchannelization, thus increasing system gain and improving indoor penetration; the use of adaptive antenna system (AAS) and multiple input multiple output (MIMO) technologies to improve coverage; the introduction of a downlink subchannelization scheme, enabling better coverage and capacity tradeoff. This brings potential benefits in terms of coverage, power consumption, self-installation, frequency reuse, and bandwidth efficiency. The 802.16e standard encompasses five Quality of Service classes for different types of traffic/applications. 

In particular, for medical applications in emergency areas it is important to have an easy setup of the infrastructure. At the same time, QoS is critical in medical applications, thus proper prioritization and scheduling policies should be adopted in order to enable reliable and high-quality transmission of possibly critical medical data. In [11] resource allocation is used to prioritize different type of connections (e.g., an emergency connection must have higher priority than a followup connection) over a IEEE 802.16e network. An admission control scheme is used to reserve radio resources for higher priority connections and avoid congestion. Three types of connections in the network are considered: connections from ambulances, clinics, and followup patients.

WiMAX has dramatically improved with respect to previous systems in terms of features which are critical for medical applications.

- High end-to-end quality;
- Robustness and Reliability: the system cannot break down under stress and the connection cannot be lost;
- Security: transmission of medical data should be secure and privacy of medical data must be preserved, medical data or patient identification cannot be disclosed indiscriminately; the fact that different health care providers have different access rights has to be considered.

However, the baseline WiMAX scheme lacks error protection beyond PHY/MAC and unequal error protection is not considered at PHY/MAC, hence video sequences can be largely affected by errors and packet losses. In order to improve the video quality, strong channel coding should be used at PHY layer. This would result in low-spectral efficiency. In addition, if unequal error protection is not available, the video quality will degrade significantly when a mobile subscriber station (MSS) experiences shadowing fading, temporal fading or interference. The idea of unequal error protection is to apply more robust channel coding to more important video content. Therefore, the MSS can at least decode some important video frames, for example, I frames and diagnostically important content.

For this reason we propose in this paper to adopt an unequal loss protection strategy at the application layer, to improve packet error resilience for ultrasound video sequences transmitted over a WiMAX system. The advantages of an unequal loss protection at the application layer are mainly the availability of detailed source information at this layer (no need to pass such information through the OSI protocol stack) and standard compatibility (PHY/MAC layers are standardized in WiMAX).

### 2.2. Wireless and Mobile Video for Telemedical Applications

Teleultrasound systems for remote diagnosis have been proposed in the last ten years [1, 2, 4, 5] given the need to allow teleconsultation when the access of the medical specialist to the sonographer is not possible.

More challenging scenarios include Ultrasound guided remote telesurgery [3] and wireless robotic teleultrasonography.

In [12], the quality of received real-time medical video sequences after transmission was just acceptable for a first diagnosis, since the available wireless technologies (2.5 G, 3 G) did not allow sufficient bandwidth for good quality video transmission. Recent studies reported by the authors in [13, 14] show the improvements achievable through the exploitation of appropriate rate-control strategies and cross-layer design over WLAN/3G systems.

Some projects and demonstrations are ongoing on multimedia telemedical application through WiMAX systems. The goal of the European IST project WEIRD [15] was the realisation of IEEE 802.16/WiMAX-based testbeds, including novel applications running on top of a WiMAX-based end-to-end architecture. The testbeds are based on real use case scenarios, including telemedicine and telehospitalization. Broadband access for medical personnel requiring high-resolution medical information in nomadic emergency camps and high-resolution video and data streaming from medical instruments were considered. The project highlighted the main challenges that WiMax still has to face in e-health applications. The goal of the “Mobile Healthcare Services” project in Taiwan [16] is to support emergency medical assistance and patient care services wherever it is required outside of a medical facility. With the assistance of high-bandwidth wireless communications (WiMAX), healthcare personnel in the field will be able to connect to critical medical resources, exchange important files, and arrange treatment, saving crucial minutes in the early treatment of patients. In Australia, with the help of Intel Australia and Airspan Networks, the organizers of the Australian Grand Prix deployed a WiMAX network to improve communication flow between the on-site trauma unit and medical specialists at the Alfred Hospital three kilometers away. Auto racing events require a medical team capable of attending to the steady stream of injuries incurred by the drivers, mechanics, and other personnel throughout the competition. The trackside trauma facility was provided with high-speed wireless connection, linking the on-site medical staff with their counterparts in a hospital three kilometers away. The WiMAX network eliminated the need for the 20-minute trips previously required to manually transport radiology images, test results, and other medical information. Furthermore, wireless web cameras installed at the remote site allowed medical staff in the field to run real-time video consultations and patient reviews with their colleagues in the hospital. The project focused on medical images and ambient video, not on medical video sequences. Further information on similar projects is provided in [17, 18].

The goal of most of the aforementioned projects is/was to demonstrate the transmission of medical data over a standard network, with no effort to tailor the characteristics of the transmission system to the specificity of the transmitted data.

One of the first works addressing the need of taking the specific characteristics of the medical application into account in the design of the transmission system was [19], presenting the design of a mobile teletrauma system using 3G Networks.

The importance of considering a specific cross-layer strategy designed with the goal of maximizing the diagnostic quality of the received information was first identified in [20] and then also addressed in [13, 14, 21, 22]. A recent work in this direction is also [23], where adaptive transmission of medical images and video is addressed using scalable coding and context-aware wireless medical networks. The authors propose a wavelet-based scalable coding scheme and context information is addressed here as the information on the patient state (normal/urgent). This work focused on teledermatology, MRI, and ambient video (no ultrasound).

### 2.3. Region-of-Interest Coding

Since most networks deal with a limited amount of bandwidth, scaling techniques are introduced to send less data over the network with as little inconvenience as possible for the user. One of these techniques is region-of-interest coding (region of interest (ROI)). ROI divides an image into multiple parts, the most important part typically being the one the user is observing, called the ROI.

ROI coding can be used to encode objects of interest with a higher quality, whereas the remainder of the image can be regarded as background information and can be encoded more coarsely. The advantage of this method is that the image parts that the viewer is looking at can be transmitted with a higher quality. The result is that the overall viewing experience remains satisfactory, while the transmission can be performed at lower bitrates.

Another advantage of ROI-coding is that ROIs can be transmitted first. This can be realized by the use of slices (e.g., if slice-group-0 is transmitted first, by placing the ROI in slice-group-0, it should arrive first at decoder side). When network congestion occurs, the probability of having a frame that contains at least something the viewer most likely wants to see, is higher with ROI coded imagery than without ROI.

The ROI can be defined by the user (e.g., clinician) by means of a mouse click, by making use of an eye tracking device or can be predicted, based on content recognition algorithms.

Medical video sequences typically consist of an area which is critical for the diagnosis and a surrounding area which provides the context, but is not critical for the purpose of the diagnosis. ROI coding appears thus as a natural methodology for medical video and ROI definition can be performed according to contextual information, either automatically or by the clinician.

Different image and video coding standards enable ROI definition, under different names. The image compression standard JPEG2000 [24] allows both the definition of a ROI of regular shape and defined by the user through a mask. The MPEG4 standard [25] proposed for the first time the concept of “objects” independently manipulated in a video sequence.

In the more recent H.264 standard [26, 27] there are several different models to implement ROI-coding, all making use of slice groups.

Indeed, the concept of ROI is not often exploited in the design of compression and transmission strategies. Reference [28] presents an implementation of multiple region-of-interest models in H.264/AVC. Compression only is addressed here. Reference [29] presents a cost-distortion optimized unequal error protection for object-based video communications, with the goal of optimizing the global distortion of each image by adapting the transmission power to provide a different protection to shape and texture information. This approach is probably the most similar to our one, although the authors focused on power adaptation (physical layer), while we propose unequal loss protection at the application layer. Furthermore, the authors did not consider medical video and did not exploit relevant context information. They exploited the “object” tool in the MPEG-4 standard, while we rely on the FMO tool in the H.264 standard for the identification of regions of interest. Reference [30] presents a region-based rate control and bit allocation for wireless video transmission. Reference [31] exploits the concept of ROI and contextual information for context-aware multi-lead ECG compression based on image codecs.

The following section describes a useful way to implement ROIs in the H.264 video coding standard.

### 2.4. Flexible Macroblock Ordering (FMO)

The H.264 standard [26, 32] includes three profiles (baseline, main, and extended), each having a different set of functionalities. The baseline profile was designed primarily for low-cost applications with reduced computational power. This profile is mainly used in mobile applications. The most relevant functionalities in the baseline profile are FMO and Arbitrary Slice Ordering (ASO). Both techniques are used for manipulating the decoding order of the macroblocks in the picture.

**Figure 1:**
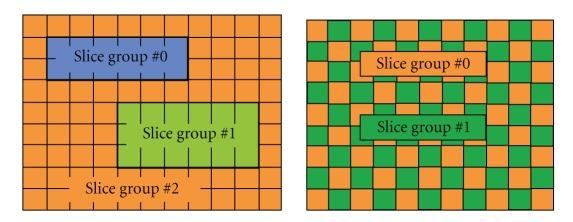
Examples of FMO patterns.

A video frame consists of macroblocks which can be grouped into slices. A slice contains at least one macroblock and it can include all the macroblocks in the video frame. Using FMO, groups of macroblocks consisting of one or more slices, known as slice groups, are formed according to a specific strategy. FMO was mainly developed with the goal of improving error concealment. The FMO mode, in conjunction with advanced error concealment methods applied at the decoder, maintains the visual impact of the losses at a low level even at loss rates up to 10%. Apart from predefined patterns, fully flexible macroblock ordering (explicit mode) is also allowed, where the macroblock classification can be changed dynamically throughout the entire video sequence based on the video content. Examples of slice groups obtained through the FMO tool are reported in Figure 1.

The idea behind FMO is that if a slice gets corrupted, and the macroblocks within this slice are dispersed across the frame, it will be easier to conceal the lost macroblocks than in the case they are contiguous.

However, according to our experience, in the case of medical video error concealment is not necessarily beneficial, since it may hide important irregularities present in the original video.

For this reason, in this paper we consider FMO as a means to perform ROI implementation in H.264 and not with the purpose of error concealment.

The standard includes seven modes to map macroblocks (MBs) to a slice group and we will consider in the following the explicit mode (Type 6), allowing the user to associate each of the macroblocks to a slice group independently. The pattern information is included in the Picture Parameter Set (PPS).

The FMO tool has already been used by a few authors for the purpose of ROI definition and unequal error protection. In [33] the authors propose a transcoding scheme to perform unequal error protection based on the information available at the output of the entropy coder. Unequal error protection is performed here in the transform domain and context/content information is not considered for the unequal error protection strategy: the most relevant macroblocks in each frame are selected solely based on the distortion introduced at the decoder if the macroblock is lost. In [34] a novel technique to represent ROIs using FMO is proposed, together with a rate control technique to improve the picture quality in ROI. In [35] the importance of every macroblock is calculated based on its influence on the current frame and future frames and macroblocks with the highest impact factor are grouped together in a separate slice group using the flexible macroblock ordering feature of H.264/AVC. The authors suggest that their results could underpin the design of an unequal error protection strategy.

### 2.5. Unequal Error Protection and Cross-Layer Design for Wireless Video Transmission

Due to the characteristics of video coding methodologies and standards [27, 36], it has been shown that joint source and channel coding/decoding techniques (JSCC/D) are beneficial to wireless video transmission [21]. Although source coding and channel coding are usually treated separately, JSCC/D techniques allow improvements in end-to-end video quality through the joint design of source coding on one side and channel coding and modulation strategies on the other. More in general, the different layers of the standard transmission protocol stack can be jointly designed, according to a paradigm that becomes to be known as cross-layer design [37]. Despite the demonstrable advantages in end-to-end video quality, there are few studies addressing the use of the cross-layer and JSCC/D approach for mobile telemedical applications [13, 14, 20, 21].

Examples of cross-layer methodologies include: rate control [38, 39], to adapt the source coding rate to the network and channel conditions; rate-distortion optimized streaming of packetized media [40]; unequal error protection. Since video packets may contribute differently to the overall video quality, unequal error protection (UEP) [41] is a natural way of protecting transmitted video data. The idea is to allocate more resources to the parts of the video sequence that have a greater impact on video quality, while spending less resources on parts that are less significant.

In [42], an unequal error protection scheme based on rate-compatible punctured convolutional codes is proposed for digital audio. In [43], a priority encoding transmission scheme is proposed to allow a user to set different priorities of error protection for different segments of the video stream. This scheme is suitable for MPEG video, in which there is a decreasing importance among I, P, and B frames. In general, error protection can come from various sources such as forward error correction (FEC), retransmission, and transmission power adaptation. In [44] the authors proposed an UEP scheme for MPEG4 video where different packet partitions were protected with different channel codes. The MPEG4 *data partitioning* tool was exploited and a criterion was proposed in order to avoid passing the otherwise necessary side information about partition lengths. Unequal error protection is also possible taking modulation into account, for example, through appropriate bit allocation over the different subcarriers in multicarrier modulation [45]. An UEP scheme based on different priorities of MPEG4 objects is presented in [29] where shape and texture data are transmitted over different service channels.

In this paper, unequal error protection is performed at the application layer through erasure codes; on one side UEP at the application layer keeps compatibility with the WiMAX standard, since MAC/PHY layers do not require modifications; on the other side, the use of erasure codes allows the recovery of lost packets at the application layer, where the use of bit-error correction codes would be useless, since lower layer protocols remove packets with erroneous bits, unless MAC-lite [46]/UDP-lite protocols are used to allow packets with erroneous bits in the payload to reach the application layer.

## 3. Problem Formulation and Proposed Transmission Scheme

### 3.1. ROI Detection

**Figure 2:**
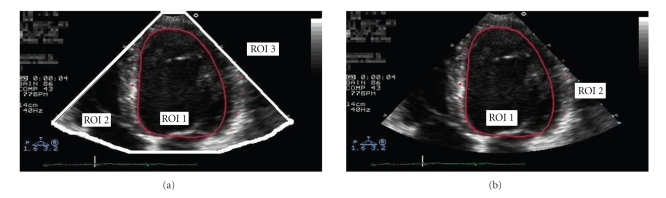
(a) Cardiac ultrasound image with ROIs (manually selected) highlighted. Three regions of interest, (b) Cardiac ultrasound image with ROIs highlighted. Two regions of interest.

This section presents a detailed formulation of the problem. The reader can refer to Table 1 for a summary of the symbols used. Ultrasound scanners produce conical images where the actual image acquired by the probe sensor is a fan-shaped window over a black background including patient data and in some cases the associated ECG waveform (see Figures 2(a) and 2(b)). The fan-shaped window is the diagnostically useful area and it typically occupies 50%–60% of the area of the full image [47]. The actual size of this fan area in a given ultrasound clip depends on the machine and its settings. After detection of three key points, this area can be modeled as the sector of a circle centered in (*a*, *b*) and with radius *c*, that is, with equation

(1) $$\begin{array}{c} {\left(x-a\right)}^{2}+{\left(y-b\right)}^{2}=c^{2}. \end{array}$$

The automatic detection of the fan area is not trivial, as the position and size of it varies in different frames and from clip to clip. Although a fan-shaped mask can be detected for each frame, we assume the fan area is uniform across all frames. We therefore construct a fan-shaped mask by finding the union of the individual masks identified. It is possible to adopt a similar procedure for clip-to-clip variations, by identifying a “universal” mask.

With the purpose of a context-aware design of the compression and transmission scheme, we identify three ROIs in each ultrasound video sequence (see Figure 2(a)).

ROI 1: Diagnostically most important area identified by the clinician (see, e.g., Figure 2(a));
ROI 2: Fan-shaped sector (see Figure 2(a));
ROI 3: Black background with patient data and in some cases the associated ECG waveform.

In the following, we will also consider ROI 2 and ROI 3 jointly processed, as in Figure 2(b).

In particular, ROI 1 is selected by the medical specialist according to context information such as type of examination and a priori knowledge on the disease to diagnose. ROI 2 can be selected automatically.

We consider two alternative options for compression and transmission of ROI 3.

- We assume we extract the information in the background prior to transmission. Information in the background is typically text data, for example, about the patient, the instrument used in the examination, and the section of the organ visualized. The associated ECG wave can also be displayed in the background area, with the ECG sample corresponding to the visualized image highlighted with a bar in the waveform. This information can be extracted prior transmission and both text and the ECG waveform can be separately compressed. When DICOM standard is used, such information can easily be separated from the rest of the image.
- We do not extract such information from the background prior to transmission and we transmit ROI 3 as a separate ROI or in the same transmission class as ROI 2.

In the first option, data and ECG waveform are separately encoded. When there is no requirement for high resolution for the diagnosis of a specific disease, ECG waveform is typically sampled at 360 Hz with a resolution of 11 bits per sample. In some cases the information from different (up to eight) channels obtained from different leads is needed. The waveform of a single channel occupies 360 samples/s × 11 bits/sample = 3960 bits/s ≈ 4 kbits/s without compression (for eight channels—12 leads—the rate is about 32 kbps). A channel can be compressed with acceptable quality down to 400 bits/s = 50 Bytes/s (see, e.g., [31] and references cited therein).

When such information is removed and separately encoded, and application layer FEC is adopted, we propose we embed such information in the padding bits needed to have a regular code structure (see Section 3.4).

Note that synchronization between ECG data and ultrasound images is important, in order for the specialist to correlate the visualized image with the corresponding wave in the ECG signal. The ECG signal provides context information to the medical specialist. For instance, it is essential for a specialist to synchronize the measurement of the diameter of vessels to the R-wave spikes in the ECG trace, to eliminate the effects of periodic changes in diameter caused by the normal changes in blood flow with every heartbeat.

### 3.2. Source Model

We assume that we compress our medical video sequences according to the H.264 video coding standard, with the aid of the Flexible Macroblock Ordering (FMO) tool for encoding separately the different ROIs. We also assume that each ROI *r* in a frame *f* is composed of *I*_*f*,*r*_ slices. We assume that different quantization parameters are adopted for each *m*-th slice for ROI *r*, *r* = 1,2, 3, in frame *f*, that is, quantization parameters are *μ*_*f*,*r*,*m*_.

The transmission channel is characterized by a set of parameters (such as packet loss rate, loss burst length, etc.) as specified in Section 3.3. For the sake of generality, we denote the service class associated to *m*th slice for ROI *r* in frame *f* as *π*_*f*,*r*,*m*_, with *r* = 1,2, 3 in our case. A service class can be intended as a QoS class provided by the underlying network or as a level of protection provided by (possibly unequal) forward error correction. The corresponding probability of packet loss and loss burst length (in packets) are denoted as *ρ*(*π*_*f*,*r*,*m*_) and *ℒ*(*π*_*f*,*r*,*m*_). The transmission rate is *R*(*π*_*f*,*r*,*m*_).

The total transmission time per frame can be calculated as

(2) $$\begin{array}{c} T_{f,\text{tot}}=\overset{r_{\text{tot}}}{\underset{r=1}{\sum }}‍\;\;\overset{I_{f,r}}{\underset{m=1}{\sum }}\left[\frac{B_{f,r,m}\left(\mu_{f,r,m}\right)}{R\left(\pi_{f,r,m}\right)}\right], \end{array}$$

where *B*_*f*,*r*,*m*_ represents the encoding bits for slice *m* in ROI *r* and *r*_tot_ is the number of ROIs in the frame. In our case *r*_tot_ = 3. Note that we do not consider here automatic retransmission request (ARQ) techniques and processing time for FEC is neglected.

In the example results reported in Section 4, we consider the case where we keep quantization parameters constant in a session and we select different service classes for different ROIs through a group of pictures GOP-by-GOP unequal error protection scheme at the application layer. In this case the model simplifies as follows. *μ*_*f*_: quantization parameter for frame *f*; *π*_*f*,*r*_: service class for ROI *r* in frame *f*; *ρ*(*π*_*f*,*r*_): corresponding probability of packet loss; *L*(*π*_*f*,*r*_): corresponding mean burst length.

The total transmission time per frame can be calculated as

(3) $$\begin{array}{c} T_{f,\text{tot}}=\overset{r_{\text{tot}}}{\underset{r=1}{\sum }}\left[\frac{B_{f,r}\left(\mu_{f}\right)}{R\left(\pi_{f,r}\right)}\right] \end{array}$$

and per GOP:

(4) $$T_{\text{GOP},\text{tot}}=\overset{f_{\text{tot}}}{\underset{f=1}{\sum }}T_{f,\text{tot}}.$$

### 3.3. Channel Model

We model here the loss pattern as a two-state Gilbert channel. The Gilbert two-state channel model [48] has been widely used in the literature to represent packet loss patterns in wireless fading channels. Two states are considered to represent good and bad channel states in terms of packet errors. 

**Figure 3:**
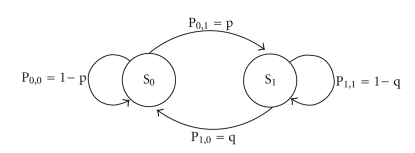
Gilbert channel model.

 Such a model, depicted in Figure 3, is completely specified by two parameters: the probability of packet loss *p*_*E*_ and the mean burst length *L*_*B*_. By denoting with *S*_0_ the packet error free state and *S*_1_ the packet error state, the channel state transition probability matrix **P** has elements *P*_*i*,*j*_ such that,

(5) $$P_{i,j}=P\left[S\left(k\right)=S_{j}\;|\;S\left(k-1\right)=S_{i}\right];\;i,j\in \left\{0,1\right\}$$

representing the transition probability from state *S*_*i*_ at *t*_*k*−1_ to state *S*_*j*_ at *t*_*k*_. 

 In particular:

(6) $$\begin{array}{c} q=P\left[S\left(k\right)=S_{0}\;|\;S\left(k-1\right)=S_{1}\right],\\ p=P\left[S\left(k\right)=S_{1}\;|\;S\left(k-1\right)=S_{0}\right]. \end{array}$$

The transition matrix is given by

(7) $$P=\left(\begin{array}{cc} 1-p & p\\ q & 1-q \end{array}\right)$$

and it is

(8) $$\begin{array}{c} p_{E}=P\left[S\left(k\right)=S_{1}\right]=\frac{p}{p+q},\\ L_{B}=\frac{1}{q}. \end{array}$$

### 3.4. Unequal Error Protection

We present in the following our application layer unequal error protection strategy. The use of Reed-Solomon (RS) codes is described first, the global UEP strategy adopted follows.

#### 3.4.1. RS Codes

We consider the use of Reed-Solomon (RS) codes for application-layer FEC. When FEC is used at the application layer, it is necessary to apply erasure codes across video packets; the WiMAX MAC layer discards the whole MAC frame in the event of an error, that is, the erroneous frame at the receiving MAC is never passed on to the higher layer. Therefore, if RS coding is applied within a single packet at the application layer, the erroneous packet will not be available for error detection or correction at the application layer.

Similar to [49], we apply RS coding across packets using an interleaver, that is, *K* slices each of length *L*_*m*_ bits are buffered at a matrix interleaver. The first symbol from each of the *K* slices is sent through an (*N*, *K*) RS coder resulting in *N* − *K* parity symbols, each of which forms the first symbol of the *N* − *K* parity packets. Note that the symbol size in bits depends on the selected value of *N*, that is, *c* = log _2_(*N* + 1). This is repeated for the whole slice, resulting in *N* − *K* parity packets each of length *L*_max _ generated by the RS encoder. Note that actually the slice lengths *L*_*m*_ are not exactly identical and padding bits are needed to obtained equally long packets of length *L*_max _.

Each video or parity packet is transmitted via RTP/UDP/IP and an 802.16e MAC frame; if this frame is discarded at the receiving 802.16e MAC layer due to channel errors, this results in a symbol erasure at the RS decoder in the application layer. The RS decoder at the application layer can correct up to *N* − *K* packet losses out of *N* packets over which the RS coding was applied.

This FEC scheme introduces delay due to two events. First, the interlacing operation requires that *K* slices are accumulated to begin the RS(*N*, *K*) coding operation. Second, once *K* packets are available, generating the redundant packets by applying the RS code involves data processing delay. Due to the high hardware speeds currently available and the possibility to perform encoding in parallel; the latter delay is very limited and we can only consider the time delay involved in having to wait for *K* packets.

Note that, since the RS code is systematic, it is not necessary to buffer packets to form RS codewords, but the information symbols can be transmitted directly if a local copy is kept to form the parity check symbols. These computed parity check symbols can then be sent immediately after the information symbols, eliminating interlacing delay at the transmitter. The total interlacing delay would then be the delay at the receiving end alone.

Every data block has its own block sequence number, which is useful at the receiver side, since it provides the RS decoder with the position of the lost block. The RS decoder can then recover up to (*N* − *K*) lost blocks with this position information instead of recovering (*N* − *K*)/2 lost blocks without the position information. The residual loss probability in case of independent packet losses is

(9) $$p_{L}=\frac{1}{N}\overset{N}{\underset{i=t+1}{\sum }}i\left(\begin{array}{c} N\\ i \end{array}\right)p_{E}^{i}{\left(1-p_{E}\right)}^{N-i},$$

where *t* = *N* − *K* when erasures are considered and information on the position of the erasures is available. For the adopted Gilbert-model the probability of packet error and burst length after FEC can be calculated following the analysis in [50].

If at least *K* out of *N* packets are correctly received, the underlying video information can be correctly decoded. Otherwise, none of the lost packets can be recovered by the receiver. This provides resiliency against a maximum packet loss rate of *p* = (*N* − *K*)/*N* when considering that even FEC packets may be affected by loss. Thus, based on averaged packet loss rate (*p*_*E*_) measurements such as that provided by RTCP feedback, it is possible to dynamically adjust the redundancy amount *h* = *N* − *K* as *h* = *p*_*E*_ *K*/(1 − *p*_*E*_). When a decoding failure happens, there are (*N* − *i*) < *K* correctly-received packets including both video and parity packets possibly. We utilize these video packets if there is any for the video decoding; on average, (*K*/*N*)(*K* − *i*) packets out of (*N* − *i*) correctly-received packets should be video packets.

#### 3.4.2. Context Adaptive UEP Strategy

We propose to provide a high protection to the most significant ROI for the purpose of diagnosis (ROI 1) and a lower protection to ROI 2 and the background. Patient data/ECG can either be transmitted as data and compressed ECG in padding bits of ROI 1 and thus strongly protected, or transmitted in ROI 2.

We propose RS coding is performed GOP by GOP; an RS block will include data from no more than one GOP.

For the selection of the RS block size, the erasure correction capability of the code and the slice size have to be defined first. The selection of slice size *L*_*f*,*r*,*m*_, when slices are not separated in smaller packets at lower layers, is linked to the network and channel characteristics. In the following we will assume that each ROI is encoded in one slice of length *L*_*f*,*r*_. We build an RS block structure of size *K* × *L*_max_, where rows are made of symbols from slices and padding bits to reach the length *L*_max_, and columns, with data in groups of bits (RS symbol), represent the RS data blocks. *L*_max _ is a value which is fixed GOP by GOP depending on the size of the slices in the GOP. The block is simply built by arranging the slices (+ padding bits) as the rows of the RS block until either the suggested value of *K* rows is reached or the slices belonging to a GOP are terminated. After RS coding the structure has size *N* × *L*_max _ due to the presence of *N* − *K* parity packets.

Note that, with the assumptions above, the MAC PDU size is

(10) $$L_{\text{PDU}}=L_{\text{MAC}.\text{header}}+L_{\text{CRC}}+L_{\text{RoHC}.\text{header}}+L_{\max\;}.$$

The selection of the coding rate *K*/*N* depends on the characteristics of the channel/the network of the video sequence and of context information and the rate could be adapted dynamically GOP-by-GOP in order to adapt to the conditions of the network.

Instead of considering models for the impact of losses in the different regions on the global distortion, as typically done, in this case we give priority to context information for taking decisions on the protection rate, the relative importance of the region of interest with respect to the background is different for different types of examinations and we propose that this weight is provided by the clinician and considered for the selection of the protection rate of the different ROIs.

After application layer unequal error protection, the total number of bits per frame is

(11) $$\begin{array}{c} B_{f,\text{tot}}=\overset{r_{\text{tot}}}{\underset{r=1}{\sum }}\left[\frac{B_{f,r}\left(\mu_{f}\right)}{\alpha R_{C}\left(\pi_{f,r}\right)}\right] \end{array}$$

and per GOP

(12) $$B_{\text{GOP},\text{tot}}=\overset{f_{\text{tot}}}{\underset{f=1}{\sum }}B_{f,\text{tot}},$$

where the factor 0 < *α* < 1 takes into account the overhead due to padding bits in the RS code matrix organization, and *f*_tot_ is the number of frames in the GOP.

#### 3.4.3. Error Concealment

It is common practice in video transmission to “conceal” the effect of errors at the receiver side by, for instance, interpolating from neighbouring data in time and space. In the medical field, this practice may not be desirable when a medical doctor is performing a diagnosis. Such concealment practice could be misleading since in this case the specialist cannot factor into his or her decision an awareness of missing and potentially important data.

For this reason, we propose that concealment is applied seamlessly only in ROI2 and ROI3 in order to smooth the not diagnostically important ROIs. Although concealment is applied in ROI1, we propose to inform the specialist that a specific MB has been concealed by highlighting concealed MBs in the portion of the video frame belonging to ROI1. It is in fact important that the specialist can assess his/her confidence on the diagnosis.

## 4. Implementation and Results

### 4.1. Simulation Scenario

The ultrasound video clips used in our experiments are cardiac ultrasonography sequences, partly collected from a Hospital and partly from public databases.

The acquired medical video sequence is encoded according to the H.264 standard [36] with the parameters reported in Table 2.

Groups of slices are organized with the aid of the flexible macroblock ordering tool, in order to have separate groups of slices for different ROIs. Information about the shape of the different ROIs is stored in the Picture Parameter Set.

The encoded image stream is then encoded through RS codes and delivered via RTP/UDP/IP.

We assume robust header compression (RoHC) is adopted to reduce the overhead due to packetization headers and that RTP/UDP/IP headers are compressed via RoHC to three bytes. 

 The main Radio Access Network parameters of the reference testbed [51] are shown in Table 3. Measurement conditions and measurement results from [51] are reported for convenience in Tables 4 and 5, respectively.

 We consider a vehicular environment, in order to simulate ultrasound video transmission to/from an ambulance. This is the case where immediate access to ultrasound examinations located in the hospital database is needed, and where the examination is performed in an ambulance through a portable ultrasonographer and the relevant video stream is transmitted in real time to the specialist in the hospital. 

 Focusing on the latter scenario, we consider uplink data transmission. In particular the Gilbert channel model parameters are selected according to the measurements in Table 5. The mean packet loss rate is set to *P*_*L*_ = 10^−1^. The measurements only report the maximum packet-loss burst length. By assuming a geometric distribution for the burst length, we estimate the mean packet-loss burst length as *L*_*B*_ = 5; in this case according to the geometric distribution the probability of having a packet-loss burst length higher than the measured maximum value is of the order of 10^−4^. Note that measurements are done by transmitting a number of packets of the order of 10^4^.

 We compare the following strategies for application layer (unequal) error protection (see Table 5).

1. No application layer protection; in this case all the available bitrate is used for representing the video sequence.
2. Application layer equal error protection (EEP): in this case a higher protection is uniformly provided to the bitstream, resulting in a higher robustness in bad channel/network conditions, but in a reduced global quality when channel/network conditions are good. RS(31,23).
3. Application layer ROI-based unequal error protection: similar as in the case above, this scheme results in a higher robustness in bad channel/network conditions, but in a reduced global quality when channel/network conditions are good. In this case, however, the redundancy is exploited to protect the most important information from the point of view of the diagnosis and an improved quality in terms of probability to perform a correct diagnosis is expected also when channel conditions are bad.
4. Application layer ROI-based and prediction-based unequal error protection; this scheme results in a higher robustness in bad channel/network conditions, but in an even more reduced global quality when channel/network conditions are good. In this case, the redundancy is exploited to protect the most important ROI from the point of view of the diagnosis and the most important information for motion compensation prediction (I frames). An improved quality in terms of probability to perform a correct diagnosis is expected also when channel conditions are bad.

### 4.2. Performance Evaluation Metrics

In medical applications, the target of the optimization of the transmission system should not be the minimization of distortion in terms of mean square error (or equivalently the maximization of the peak signal-to-noise ratio, PSNR), but the maximization of the probability of performing a correct diagnosis based on the received video sequence. Although not designed for this purpose, according to preliminary studies [13, 52] the structural similarity metric (SSIM) [53] better meets this criterion and for this reason we consider in this paper SSIM in addition to the well-known PSNR. Although the performance assessment of such a scheme should be done through subjective metrics, results are presented in terms of the aforementioned well known objective metrics in order to allow easy comparison with results obtained by other authors and to give an indication of the local distortion achieved in different ROIs.

 We consider local distortion as in the following:

(13) $$\begin{array}{c} {\text{MSE}}_{\text{ROI}r}=\frac{1}{N_{r}}\overset{N_{r}}{\underset{i=1}{\sum }}{\left(x_{i}-y_{i}\right)}^{2},\\ {\text{PSNR}}_{\text{ROI}r}=10\;{\log\;}_{10}\frac{255^{2}}{{\text{MSE}}_{\text{ROI}r}}, \end{array}$$

where *N*_*r*_ is the number of pixels in ROI; *r*,  *x*_*i*_ and *y*_*i*_ represent the luminance of pixel *i* in the original and in the corrupted frame, respectively.

 We then consider a slightly modified version of the SSIM metric in [53]. The SSIM index, as shown in (14), can be written as the product of three independent contributions, representing the luminance information, the contrast information, and the structural information. With **x** and **y** indicating image signals in the reference and received image,

(14) SSIM(x, y)=l(x, y)·c(x, y)·s(x, y),

where the luminance comparison is represented by the term

(15) $$l\left(x,\;y\right)=\frac{2\mu_{x}\mu_{y}+C_{1}}{\mu_{x}^{2}+\mu_{y}^{2}+C_{1}}$$

and for the contrast comparison

(16) $$c\left(x,\;y\right)=\frac{2\sigma_{x}\sigma_{y}+C_{2}}{\sigma_{x}^{2}+\sigma_{y}^{2}+C_{1}}.$$

The structural comparison term *s*(*x*, *y*) is

(17) $$s\left(x,\;y\right)=\frac{\sigma_{xy}+C_{3}}{\sigma_{x}\sigma_{y}+C_{3}}.$$

In the expressions above *C*_1_, *C*_2_, and *C*_3_ are appropriate constant values. At each coordinate, the SSIM index is calculated within a local window. As in [53], we use a 11 × 11 circular-symmetric Gaussian weighting function *w*_*i*_, with standard deviation of 1.5 samples, normalized to sum to unity: ∑_*i*_ *w*_*i*_ = 1. The statistics are thus defined as

(18) $$\begin{array}{c} \begin{array}{c} \mu_{x}=\frac{1}{N}\overset{N}{\underset{i=1}{\sum }}w_{i}x_{i}, \end{array}\\ \sigma_{x}^{2}=\frac{1}{N-1}\overset{N}{\underset{i=1}{\sum }}w_{i}{\left(x_{i}-\mu_{x}\right)}^{2},\\ \sigma_{xy}=\frac{1}{N-1}\overset{N}{\underset{i=1}{\sum }}w_{i}\left(x_{i}-\mu_{x}\right)\left(y_{i}-\mu_{y}\right). \end{array}$$

We then define the MSSIM metric for ROI*r* as

(19) $$\begin{array}{c} {\text{MSSIM}}_{{\text{ROI}}_{r}}\left(X_{r},Y_{r}\right)=\frac{1}{M}\overset{M}{\underset{j=1}{\sum }}\text{SSIM}\left(x_{r,j},y_{r,j}\right), \end{array}$$

where *M* is the number of local windows in ROI *r* and *j* is the local window index.

### 4.3. Numerical Results

Numerical results obtained in the conditions described above, and summarized in Table 6, are reported in Table 7. PSNR and SSIM values are average values over the different frames of the sequence. Local PSNR and SSIM values are also reported, as defined in Section 4.2.

Note that the quality of the diagnostically important region of interest is lower than the quality of the background in the unprotected case, due to the different complexity and the use of the same quantization parameter for the different ROIs.

A uniform protection scheme at the application layer (EEP) increases the total quality of the sequence, but it fails in sensibly increasing the quality of the most important ROI for the diagnosis. We highlight here that the schemes where FEC is applied at the application layer are compared with an uncoded scheme where a higher bitrate is adopted in source encoding, in order to allow a fair comparison.

Both the UEP schemes manage to improve the quality of ROI 1, at the expense of a slight decrease in quality in the remaining part of the images. The scheme UEP 1 provides a slightly higher quality for ROI 1, both in terms of PSNR and SSIM. Scheme UEP 2 provides an improvement of about 1 dB in PSNR with a decrease in quality with respect to scheme UEP 1 of only 0.2 dBs in ROI 1 and it can be preferable in some scenarios as also confirmed by subjective tests.

**Figure 4:**
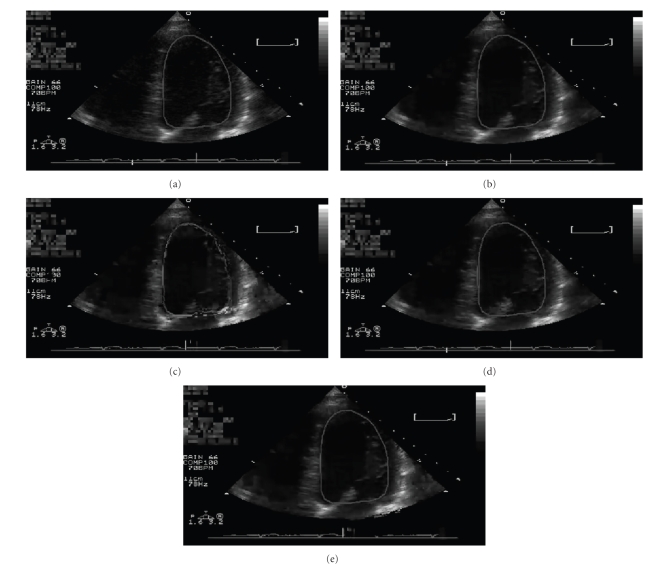
Visual results—Frame no. 44 of the test sequence. (a) Original; (b) Uncoded (Scheme 2 in Table 6); (c) EEP (Scheme 1 in Table 6); (d) UEP 1 (Scheme 3 in Table 6); (e) UEP 2 (Scheme 4 in Table 6).

Visual results are reported in Figure 4. The sequence corresponding to image [e] is the one selected by the medical specialist involved in the study as the one best keeping diagnostic quality.

## 5. Conclusion

We have proposed in this paper a context-aware transmission strategy for diagnostic-quality ultrasound video transmission over WiMAX systems. Context, in terms of regions of interest (ROI) in a specific session, is taken into account for the identification of multiple regions of interest, and compression/transmission strategies are tailored to such context information. We have presented a methodology based on H.264 medical video compression and FMO for ROI identification. Two different unequal error protection methodologies, providing higher protection to the most diagnostically relevant data, are compared. Results show that the proposed scheme allows an improvement for the diagnostic region of interest of about 3 dBs in PSNR and 0.31 in SSIM with respect to the case where such an approach is not adopted, still obtaining a small improvement in quality in the rest of the image (0.8–1.6 in PSNR for UEP 1 and UEP 2, resp.). This methodology is simple to implement and standard compatible.

## Figures and Tables

**Table 1:** Summary of important symbols used.

Symbol	Definition
*m*	Slice index
*r*	ROI index
*f*	Frame index
*L*_*f*,*r*,*m*_	Length of slice *m* in ROI *r*, frame *f*
*L*	Average slice length
*L*_max _	Max slice length
*r*_tot_	Number of ROIs
*f*_tot_	Number of frames per GOP
*I*_*f*,*r*_	Number of slices in ROI *r* in frame *f*
*N*_*f*,*r*_	Number of pixels in ROI *r* in frame *f*
*μ*_*f*,*r*,*m*_	Quantization parameter(s) for slice *m*, ROI *r*, frame *f*
*π*_*f*,*r*,*m*_	Service class
*p*_*E*_	Generic probability of packet loss
*p*_*L*_	Residual probability of packet loss after RS coding
*ρ*(*π*_*f*,*r*,*m*_)	Probability of packet loss for service class *π*_*r*,*m*_
*L*_*B*_	Generic mean error burst length (in packets)
*q*	Transition prob. lossy → lossless (Gilbert model)
*p*	Transition prob. lossless → lossy (Gilbert model)
*ℒ*(*π*_*f*,*r*,*m*_)	Mean error burst length for service class *π*_*r*,*m*_
*B*_*f*,*r*,*m*_	Encoding bits for slice *m* in ROI *r*, frame *f*
*c*	RS symbol size
*N*	RS code block length (in *q*-ary symbols)
*K*	RS data block size (in *q*-ary symbols)
*R*_*C*_ = *K*/*N*	RS code rate
*R*_*C*_(*π*_*r*,*m*_)	RS code rate for service class *π*_*r*,*m*_
*R*(*π*_*r*,*m*_)	Transmission rate for service class *π*_*r*,*m*_
*T*_*f*,tot_	Transmission time per frame
*x*_*i*_	Luminance of pixel *i* in original frame
*y*_*i*_	Luminance of pixel *i* in received frame

**Table 2:** Video coding simulation parameters.

Encoder/Decoder Parameter	Value
Encoder/Decoder	JM reference software codec Version 16.0
Profile	Baseline profile
Test sequences	Guillaume-us
No. of frames	70
Resolution	480 × 256
GOP size	15 (IPPP…)
Quantization parameters	30/33
Reference frames	1
Entropy coding	CAVLC (Content Adaptive Variable Length Coding)
Decoder error concealment	JM-FC (JM-Frame Copying)

**Table 3:** WiMAX RAN system parameters.

Parameter	Value
Channel bandwidth	5 MHz
Carrier frequency	3468.5 MHz and 3568.5 MHz
Number of subcarriers	512
Number of used data subcarriers	360
Cyclic prefix	1/4 symbol duration
Frame length	5 ms
Channel Coding	Turbo Codes
Possible modulation and coding schemes	QPSK 1/2, 16 QAM 1/2, 64 QAM 1/2
ARQ	No ARQ scheme
Number of BS antennas	2
BS antenna	type 4 array antenna
BS antenna height	22 m
BS antenna gain	17 dBm
BS transmission power	35 dBm
BS antenna azimuth	6° and 276°
MS antenna gain	2 dBi
MS transmission power	23 dBm

**Table 4:** Vehicular measurement parameters.

Distance to BS antenna	281 m–500 m
Scenario	Sub-Urban
Mobile speed	50 km/h
Max. channel bandwidth	500 kbps
Packets per second	38

**Table 5:** WiMAX measurement results considered for simulation.

MIN Delay [s]	MAX Delay [s]	Average Delay [s]	Max Packet Loss Burst Length	Packet Loss [%]
0.017273	0.184955	0.047212	28	10.14

**Table 6:** Application-layer unequal error protection strategies adopted.

		Source bitrate	ROI 1 frames I	ROI 2 frames I	ROI 1 frames P	ROI 2 frames P
1	No application layer FEC	480 kbps	—	—	—	—
2	Application layer EEP	300 kbps	RS(31,23)	RS(31,23)	RS(31,23)	RS(31,23)
3	Application layer UEP based on ROIs	300 kbps	RS(31,16)	—	RS(31,16)	—
4	Application layer UEP based on ROIs and prediction	300 kbps	RS(31,22)	RS(31,22)	RS(31,22)	—

**Table 7:** Video quality results.

	UNCODED			EEP			UEP 1			UEP 2		
	Overall	ROI 1	ROI 2	Overall	ROI 1	ROI 2	Overall	ROI 1	ROI 2	Overall	ROI 1	ROI 2
PSNR (dB)	33.11	30.94	34.01	34.63	30.92	36.12	34.15	33.26	33.85	34.95	33.06	35.65
SSIM	0.89511	0.81503	0.89953	0.89881	0.81443	0.91568	0.89845	0.85342	0.90887	0.90424	0.84647	0.9144
